# Exploring Health-Seeking Behaviors Among Healthcare Workers and the General Population During the COVID-19 Pandemic: A Retrospective Quantitative Study

**DOI:** 10.1177/11786329251316698

**Published:** 2025-02-06

**Authors:** Gabriela Castañeda-Millán, Alexia M. Haritos, Edris Formuli, Maryna Mazur, Kishana Balakrishnar, Bao-Zhu Stephanie Long, Behdin Nowrouzi-Kia

**Affiliations:** 1 Department of Occupational Science and Occupational Therapy, Temerty Faculty of Medicine, ReSTORE Lab, University of Toronto, Toronto, ON, Canada; 2 Universidad Nacional de Colombia, Bogotá, Colombia; 3 Department of Psychology, University of Toronto Scarborough, Scarborough, ON, Canada; 4 Department of Health and Society, University of Toronto Scarborough, Scarborough, ON, Canada; 5 Krembil Research Institute-University Health Network, Toronto, ON, Canada; 6 Centre for Research in Occupational Safety & Health, Laurentian University, Sudbury, ON, Canada; 7 Institute for Mental Health Policy Research, Centre for Addiction and Mental Health, Toronto, ON, Canada

**Keywords:** Mental health, healthcare workers, help-seeking behavior, Canada, barriers, sociodemographic factors, predictors, mental health services

## Abstract

**Background/objectives:**

Mental health issues are prevalent among healthcare workers, but help-seeking behavior in this groups remains under-researched. The purpose of this study was to explore predictors of and barriers to mental health help-seeking among healthcare workers in Canada, compared to workers from other sectors.

**Design:**

This quantitative study analyzed cross-sectional data from Mental Health Research Canada (MHRC) from October 2022 to January 2024.

**Methods:**

The total sample consisted of 8,191 workers from various sectors, including 419 healthcare workers. We examined prevalence of help-seeking, barriers to accessing mental health support, and predictors of help seeking using descriptive and inferential statistics. A multivariate logistic regression analysis was performed to explore the relationship between sociodemographic factors and help-seeking.

**Results:**

Healthcare workers were more likely to seek mental help support compared to workers from other sectors (OR 1.73, 95% CI: 1.35, 2.20). Healthcare workers least likely to seek mental health support were male (OR 0.58, CI 0.52, 0.66), residing in Quebec (OR 0.49, 95% CI: 0.41, 0.59), or of older age (OR 0.40, 95% CI: 0.30, 0.52). Key barriers to mental health help-seeking identified among healthcare workers included concerns about exposure to COVID-19 (33%), preference for self-management (25%), concerns about the safety of care options (18%), and lack of knowledge on how or where to seek help (13%).

**Conclusions:**

This study provides valuable insight into the barriers and predictors of mental help-seeking behavior among healthcare workers. Findings underscore the need for workplaces to foster safe, supportive, and inclusive environments to better support healthcare workers facing mental health challenges.

## Introduction

Mental disorders are a leading cause of disability worldwide, with anxiety and depression being among the most prevalent conditions. In 2019, nearly 970 million people were affected by some form of mental disorder, and individuals with these conditions face a significantly shortened life expectancy, often 10 to 20 years less than the general population. This disparity is largely due to increased suicide risk and associated health complications.^1^

Mental health disorders substantially impact occupational functioning. In 2019, the World Health Organization (WHO) classified burnout as an “occupational phenomenon” in the International Classification of Diseases 11th Revision.^2^ Healthcare workers, in particular, experience high rates of burnout, stress, and depression primarily due to excessive workloads, long shifts, a fast-paced environment, lack of physical or psychological safety, chronic care demands, moral conflicts, perceived job insecurity, workplace harassment or lack of social support.^3^ Notably, the suicide rate has been found to be higher among physicians compared to the general population, with statistics exhibiting a 1.05 times higher rate in male physicians and 1.76 times higher in female physicians.^4^

The COVID-19 pandemic aggravated these issues, with increased workloads further deteriorating mental health conditions.^5^ During this period, the prevalence of post-traumatic stress, anxiety, depression, and burnout among healthcare workers is 49%, 40%, 37%, and 37%, respectively.^6^ Thus, promoting mental healthcare among healthcare personnel is imperative, as their mental health status not only impacts their functioning but also the care received by their patients.^3^ Despite the clear need, help-seeking behavior for mental health challenges remains low among this population. Reports indicate that around 50% of female physicians do not seek treatment despite recognizing that they meet the criteria for mental disorders.^7^ One significant barrier is stigma, which involves prejudice and negative attitudes.^8^ This stigma particularly impacts younger female healthcare workers.^9^ However, male physicians are less likely to seek mental health care than female physicians. with stigma surrounding male help-seeking being a contributing factor.^10,11^

In Canada, the situation mirrors global trends, with a high prevalence of mental disorders and worsened mental health following the pandemic. By 2024, self-reported anxiety and depression rates are 11% and 12%, respectively, with a 3% increase in depression. These figures vary among populations, with Alberta and Ontario showing the worst mental health outcomes. The most vulnerable groups include youth, Indigenous peoples, and members of the 2SLGBTQIA+ community.^12^ According to the WHO, anxiety and depression increased 25% globally from the COVID-19 pandemic.^13,14^

The concern of help-seeking behavior for mental health issues is becoming increasingly significant. In a population of over 40 million, 1 in 5 Canadians experience some form of mental health challenge each year.^15^ Only 15% of those affected access some form of support, and 6% feel the need for support but do not seek it.^12^ A 2020 survey reveals that only 8.2% of healthcare professionals utilized professional mental health services.^16^ Mental health risks also tend to be higher for certain types of physicians. For instance, one study found that palliative care professionals faced mental health challenges as substantial as physicians treating COVID-19 directly.^17^ Previous studies have identified factors influencing help-seeking for mental health among Canadian physicians, including professional implications, job position, quality of care, confidentiality, convenience, and social stigma.^18,19^

This study aims to identify whether Canadian healthcare workers are more or less likely to engage in health-seeking behaviors amid the COVID-19 pandemic compared to the general population. Comparing the health-seeking behaviors of healthcare workers to those of non-healthcare workers can unveil whether occupation influences such behaviors especially during times of crisis. Understanding these differences can aid in identifying barriers and facilitating health access for healthcare workers especially given their importance during major health catastrophes.

## Methodology

A cross-sectional analysis was conducted using poll data from Mental Health Research Canada (MHRC). The Mental Health Research Canada dataset contains nineteen online polls administered from April 2020 to January 2024. Poll 1 was administered in April 2020, and the final poll 19 was administered in January 2024. Each polls data collection period was approximately one to two weeks. The online survey “Understanding the Mental Health of Canadians Through Covid-19 and Beyond” had a total sample size of 62508 among Canadians aged 18 and above. The polls 1-19 overall provide insight into the mental health of Canadians from April 2020 to January 2024. The surveys were designed in accordance with ethical guidelines, ensuring that participants were informed about the purpose of the survey, their voluntary participation, and their right to withdraw at any time.

Data analysis was performed on MHRC polls 14-19, covering the time between October 2022 and January 2024. Probabilistic sampling was performed based on demographic and regional quotas to reflect Canada’s actual population. The total sample size across polls 14-19 inclusive was 8,391 Canadian workers. The data were weighted by gender, age, and region according to the most recent Census data.^20^ The sampling strategy was probabilistic, weighted by census data in terms of gender, age, and geography (i.e. province) to ensure the total sample was representative of the population as a whole. Informed consent was not sought for the present study due to the nature of the data collection and the secondary analysis of anonymized survey data.

See Table 1 for the criteria used to determine subject eligibility. The categories of healthcare workers included were Health Care—Front-line Health Care Workers caring for COVID-19 patients, Health Care—Patient-facing Health Care Workers not caring for COVID-19 patients, Health Care—Back-office Health Care Workers who are not patient-facing, and Health Care—Other. All other workers were categorized as non-healthcare workers.

**Table 1.** Inclusion and exclusion criteria.

Criteria	Inclusion criteria	Exclusion criteria
Age	18+ years of age	<18 years of age
Residency	Canadian residents	Non-Canadian residents
Employment status	Employed in either healthcare or non-healthcare sectors	Not employed

The analysis focused on seeking mental health assistance according to the sociodemographic characteristics of the workers. These included the province of residence and whether the respondent lived in a rural or urban area. Employment type was categorized into healthcare roles such as doctors, nurses, allied health professionals (including mental health professionals like psychologists, therapists, or counselors), pharmacists, technologists or technicians, and physical therapists. Age was classified into five groups: 18 to 30, 31 to 40, 41 to 50, 51 to 60, and 61 or more. Gender identity was captured as male, female, another gender identity, with the latter recognizing individuals outside the binary gender spectrum. The highest level of education completed was also considered, with respondents classified as having completed elementary or high school, college or technical/trade school or apprenticeship, university undergraduate degree, and university graduate/professional degree. Self-Identification categories included individuals who identified as part of a visible minority, which refers to racial or ethnic groups that are not part of the majority in Canada. Indigenous participants included those who self-identified as First Nations, Metis, or Inuit. Additionally, the 2SLGBTQIA+ category represented individuals who identified as Two-Spirit, Lesbian, Gay, Bisexual, Transgender, Queer, Intersex, Asexual, or as having other diverse sexual or gender identities. Other categories included those with physical impairments, refugees, or participants who did not identify with any of these categories. Total household income was grouped into ranges: no income, under $20,000; $20,000 - less than $30,000; $30,000 - less than $50,000; $50,000 - less than $80,000; $80,000 - less than $100,000; $100,000 – less than $150,000; $150,000 or more.

Seeking mental health assistance was operationalized as having received some type of support in the past year, with possible outcomes including having received help, not having received help, and feeling the need for help but not having accessed it. The study quantified these findings using prevalence percentages (i.e. number of people who sought divided by the sample size) and odds ratio.

For statistical analysis, categorical variables were described using absolute and relative frequency tables, and continuous variables were described using means and medians, depending on their parametric or non-parametric distribution. A univariate analysis was conducted using Fisher’s tests to identify differences in seeking mental health assistance according to the aforementioned variables. A binary logistic regression model was built to identify factors associated with seeking mental health assistance among healthcare workers.

Help-seeking behavior was operationalized by quantifying mental health service usage. Mental health service usage was measured by the following: “Have you accessed any kind of mental health or substance use service or resource in the past year? This includes talking to doctors or other healthcare providers, people in a group, chat or telephone service or reading self-help resources.” For the regression model, the two outcomes were as follows: “No, I have never accessed services” and “Yes, I have accessed services in the past year.” They were coded as 0 or 1.

Ethical approval was obtained via the University of Toronto Research Ethics Board. Permission was sought and approved from the Mental Health Research Canada (MHRC) for the use of the dataset.

## Results

Of the total sample, a mere 5% (*n* = 419) were employed in the healthcare field, with the rest being from other sectors (Table 2). According to the data, 57% of healthcare workers reported they did not seek mental health support last year, 28% reported seeking help, and 15% felt the need to seek help but did not. Among this last group, the most prevalent reasons for not seeking help were (1) Concerned about exposure to COVID-19 (33%), (2) Preference to manage their issues by themself (25%) (3) They did not feel safe with the care options available (18%), and (4) they did know how or where to receive help (13%). Moreover, out of the individuals who sought help, a total of 79% reported being satisfied (somewhat satisfied 34%; very satisfied 45%). See Table 3.

**Table 2.** Sociodemographic characteristics of Canadian workers.

Variable	Overall^a^	Healthcare workers^a^	Non healthcare workers^a^	*p*-Value^b^
	n = 8,391	n = 419	n = 7,972	
Healthcare sector				
Doctor	61 (15%)	61 (15%)	-	
Nurses	223 (53%)	223 (53%)	-	
Allied	135 (32%)	135 (32%)	-	
Employment status				<.001
Full time	6,511 (78%)	319 (76%)	6,192 (78%)	
1 employ part time	922 (11%)	68 (16%)	854 (11%)	
2 employ part time	187 (2.2%)	15 (3.6%)	172 (2.2%)	
Self-employ	771 (9.2%)	17 (4.1%)	754 (9.5%)	
Province				<.001
Atlantic Provinces	990 (12%)	80 (19%)	910 (11%)	
British Columbia	1,064 (13%)	45 (11%)	1,019 (13%)	
Prairie Provinces	1,898 (23%)	102 (24%)	1,796 (23%)	
Ontario	2,640 (31%)	93 (22%)	2,547 (32%)	
Quebec	1,799 (21%)	99 (24%)	1,700 (21%)	
Age				<.001
18-30	1,708 (20%)	148 (35%)	1,560 (20%)	
30-40	2,193 (26%)	144 (34%)	2,049 (26%)	
40-50	1,935 (23%)	59 (14%)	1,876 (24%)	
50-60	1,784 (21%)	49 (12%)	1,735 (22%)	
>60	771 (9.2%)	19 (4.5%)	752 (9.4%)	
Gender				<.001
Woman	3,717 (44%)	300 (72%)	3,417 (43%)	
Man	4,635 (55%)	118 (28%)	4,517 (57%)	
Other identity	39 (0.5%)	1 (0.2%)	38 (0.5%)	
Area of residence				.4
Urban	7,402 (88%)	364 (87%)	7,038 (88%)	
Rural	989 (12%)	55 (13%)	934 (12%)	
Highest level education				<.001
Elementary or High School	1,203 (14%)	8 (1.9%)	1,195 (15%)	
College or Technical	2,635 (31%)	128 (31%)	2,507 (31%)	
Undergraduate	2,898 (35%)	137 (33%)	2,761 (35%)	
Graduate	1,655 (20%)	146 (35%)	1,509 (19%)	
Household income				<.001
No income	12 (0.1%)	2 (0.5%)	10 (0.1%)	
Under $20,000	262 (3.1%)	11 (2.6%)	251 (3.1%)	
$20,000 - Less than $30,000	462 (5.5%)	16 (3.8%)	446 (5.6%)	
$30,000 - Less than $50,000	978 (12%)	28 (6.7%)	950 (12%)	
$50,000 - Less than $80,000	1,760 (21%)	75 (18%)	1,685 (21%)	
$80,000 - Less than $100,000	1,412 (17%)	84 (20%)	1,328 (17%)	
$100,000 – Less than $150,000	2,007 (24%)	103 (25%)	1,904 (24%)	
$150,000 or More	1,498 (18%)	100 (24%)	1,398 (18%)	
Self-identification				.008
Visible minority	1,584 (19%)	94 (22%)	1,490 (19%)	
Physical impairment	207 (2.5%)	17 (4.1%)	190 (2.4%)	
2SLGBTQIA+	495 (5.9%)	28 (6.7%)	467 (5.9%)	
Refugee	23 (0.3%)	3 (0.7%)	20 (0.3%)	
None of the above	6,082 (72%)	277 (66%)	5,805 (73%)	

a Frequency (%).

b Fisher’s exact test.

**Table 3.** Barriers to seeking mental health help and satisfaction with services.

Variable	Overall	Healthcare workers	Non healthcare workers	*p* ^b^
	n = 8,391^a^	n = 419^a^	n = 7,972^a^	
Have you accessed any kind of mental health or substance use service or resource in the past year?				<.001
I have never accessed services	6,265 (75%)	239 (57%)	6,026 (76%)	
Yes, I have accessed services in the past year	1,514 (18%)	119 (28%)	1,395 (17%)	
I have felt the need to access mental health services in the past year, but have not actually done so	612 (7.3%)	61 (15%)	551 (6.9%)	
Why did you not access the support of a mental health professional?				.76
Preferred to manage self	163 (27%)	15 (25%)	148 (27%)	
Concerned about exposure to COVID	128 (21%)	20 (33%)	108 (20%)	
Didn't feel safe with the care options available	86 (14%)	11 (18%)	75 (14%)	
Didn’t know how or where to get help	81 (13%)	8 (13%)	73 (13%)	
Your job interfered	34 (5.6%)	2 (3.3%)	32 (5.8%)	
Haven’t gotten around to it	34 (5.6%)	1 (1.6%)	33 (6.0%)	
Didn't have confidence in health care system / social services	29 (4.7%)	1 (1.6%)	28 (5.1%)	
Access to care was limited	15 (2.5%)	2 (3.3%)	13 (2.4%)	
Afraid of what others would think	9 (1.5%)	1 (1.6%)	8 (1.5%)	
Caretaker obligations	7 (1.1%)	0 (0%)	7 (1.3%)	
Insurance did not cover it	6 (1.0%)	0 (0%)	6 (1.1%)	
Wait times are too long	6 (1.0%)	0 (0%)	6 (1.1%)	
Couldn’t afford to pay	5 (0.8%)	0 (0%)	5 (0.9%)	
Other	5 (0.8%)	0 (0%)	5 (0.9%)	
Couldn’t find culturally sensitive care	2 (0.3%)	0 (0%)	2 (0.4%)	
Language Problems	2 (0.3%	0 (0%)	0 (0%)	
How satisfied were you with the mental health supports you received in the past year, from all sources?				.13
Very Satisfied	514 (34%)	54 (45%)	460 (33%)	
Somewhat satisfied	592 (39%)	41 (34%)	551 (39%)	
Not very satisfied	122 (8.1%)	5 (4.2%)	117 (8.4%)	
Not at all satisfied	58 (3.8%)	4 (3.4%)	54 (3.9%)	
Neutral	215 (14%)	14 (12%)	201 (14%)	
Unsure	13 (0.9%)	1 (0.8%)	12 (0.9%)	

a Frequency (%).

b Fisher’s exact test.

In accordance with employment, differences were found in help-seeking behavior for mental health support. Specifically, 33% of healthcare workers sought help while 67% did not. Whereas within other sectors, 19% of workers sought help, and 81% did not. Seeking support was the lowest in Quebec, where only 12% of people sought help. Seeking help occurred more frequently among those with higher education levels but at lower rates among those with higher incomes. It was also more frequent among the 2SLGBTQIA+ population and those with physical impairments (see Table 4).

**Table 4.** Factors associated with seeking mental health help among Canadian workers.

Variable	Unadjusted model		Adjusted model	
	OR	95% CI	OR	95% CI
Employ				
Non-Healthcare worker	—	—	—	—
Healthcare worker	2.15	1.71, 2.69	1.73	1.35, 2.20
Province				
Ontario	—	—	—	—
Atlantic Provinces	1.20	1.00, 1.44	1.17	0.97, 1.42
British Columbia	1.05	0.88, 1.26	1.09	0.90, 1.32
Prairie Provinces	1.07	0.92, 1.24	1.10	0.94, 1.29
Quebec	0.55	0.46, 0.65	0.49	0.41, 0.59
Employment status				
Full time	—	—	—	—
1 employ part time	1.02	0.85, 1.22	0.89	0.73, 1.09
2 employ part time	1.69	1.19, 2.35	1.33	0.92, 1.91
Self-employ	0.74	0.60, 0.91	0.84	0.67, 1.06
Age				
18-30	—	—	—	—
30-40	0.74	0.63, 0.86	0.83	0.71, 0.98
40-50	0.55	0.46, 0.65	0.65	0.55, 0.78
50-60	0.43	0.36, 0.52	0.54	0.44, 0.65
>60	0.32	0.25, 0.41	0.40	0.30, 0.52
Gender				
Woman	—	—	—	—
Man	0.54	0.48, 0.61	0.58	0.52, 0.66
Other identity	6.76	3.27, 15.0	3.43	1.56, 7.98
Area of residence				
Urban	—	—	—	—
Rural	0.79	0.66, 0.95	0.83	0.68, 1.00
Highest level education				
Elementary or High School	—	—	—	—
College or Technical	0.94	0.78, 1.13	0.95	0.78, 1.16
Undergraduate	1.21	1.01, 1.44	1.13	0.93, 1.37
Graduate	1.26	1.04, 1.53	1.16	0.94, 1.44
Household income				
No income	—	—	—	—
Under $^+^20,000	0.55	0.17, 1.93	0.63	0.18, 2.41
$20,000—Less than $30,000	0.37	0.11, 1.27	0.45	0.13, 1.69
$30,000—Less than $50,000	0.41	0.13, 1.40	0.56	0.17, 2.09
$50,000—Less than $80,000	0.32	0.10, 1.09	0.41	0.12, 1.53
$80,000—Less than $100,000	0.33	0.10, 1.12	0.42	0.12, 1.56
$100,000—Less than $150,000	0.32	0.10, 1.07	0.42	0.12, 1.55
$150,000 or More	0.31	0.10, 1.06	0.44	0.13, 1.64
Self-identification				
None of the above	—	—	—	—
Visible minority	1.16	1.00, 1.35	0.94	0.80, 1.09
Physical impairment	2.88	2.07, 3.97	2.81	1.99, 3.95
2SLGBTQIA+	3.49	2.85, 4.25	3.22	2.60, 3.99
Refugee1	1.48	0.42, 4.20	1.38	0.38, 4.02

OR = Odds Ratio; CI = Confidence Interval.

+ Canadian dollars.

There were some geographic differences between urban and rural healthcare workers depending on the analysis method. There was no significant difference between healthcare workers and non-healthcare workers in Table 2. There was a significant difference in seeking mental health support versus not between healthcare and non-healthcare workers in Table 5. In the regression model, a near-significant association was observed between rural and urban workers, with rural workers being less likely to seek mental health assistance in comparison to urban workers in both the unadjusted (OR 0.79, 95% CI: 0.66, 0.95) and adjusted model (OR 0.83, 95% CI: 0.68, 1.00).

**Table 5.** Mental health help-seeking among Canadian workers.

Variable	Did not seek help	Did seek help	*p*-value^b^
	n = 6,265^a^	n = 1,514^a^	
Employ			<.001
Non-Healthcare	6,026 (81%)	1,395 (19%)	
Healthcare	239 (67%)	119 (33%)	
Province			
Ontario	1,925 (79%)	497 (21%)	
Prairie Provinces	1,374 (78%)	379 (22%)	
Quebec	1,503 (88%)	213 (12%)	
British Columbia	765 (79%)	208 (21%)	
Atlantic Provinces	698 (76%)	217 (24%)	
Employment status			<.001
Full time	4,847 (80%)	1,183 (20%)	
1 employ part time	677 (80%)	169 (20%)	
Self-employ	622 (85%)	113 (15%)	
2 employ part time	119 (71%)	49 (29%)	
Age			<.001
18-30	1,075 (72%)	418 (28%)	
30-40	1,549 (78%)	444 (22%)	
40-50	1,507 (82%)	321 (18%)	
50-60	1,466 (86%)	247 (14%)	
>60	668 (89%)	84 (11%)	
Gender			<.001
Man	3,656 (85%)	646 (15%)	
Woman	2,599 (75%)	846 (25%)	
Other identity	10 (31%)	22 (69%)	
Area of residence			.012
Urban	5,481 (80%)	1,360 (20%)	
Rural	784 (84%)	154 (16%)	
Highest level education			<.001
Elementary or High School	909 (82%)	200 (18%)	
College or technical	2,050 (83%)	423 (17%)	
Undergraduate	2,126 (79%)	564 (21%)	
Graduate	1,180 (78%)	327 (22%)	
Household income			<.001
No income	7 (58%)	5 (42%)	
Under $^c^20,000	167 (72%)	66 (28%)	
$20,000—Less than $30,000	323 (79%)	85 (21%)	
$30,000—Less than $50,000	691 (77%)	203 (23%)	
$50,000—Less than $80,000	1,295 (81%)	296 (19%)	
$80,000—Less than $100,000	1,068 (81%)	251 (19%)	
$100,000—Less than $150,000	1,534 (82%)	346 (18%)	
$150,000 or More	1,180 (82%)	262 (18%)	
Self-identification			
None of the above	4,743 (83%)	984 (17%)	
Visible Minority	1,147 (81%)	277 (19%)	
2SLGBTQIA+	260 (58%)	188 (42%)	
Physical impairment	102 (63%)	61 (37%)	
Refugee	13 (76%)	4 (24%)	

a Frequency (%).

b Fisher’s exact test.

c Canadian dollars.

Factors predicting the likelihood of seeking mental health support among healthcare workers include, identifying as a gender other than man or woman (OR 3.43, 95% CI: 1.56-7.98), belonging to the 2SLGBTQIA+ community (OR 3.22, 95% CI: 2.60-3.99), and having a physical impairment (OR 2.81, 95% CI: 1.99-3.95). In addition, being a healthcare worker was associated with higher odds of seeking mental health support compared to the general population (OR 1.73, 95% CI: 1.35-2.20). Conversely, factors predicting a lower likelihood of seeking support among healthcare workers included residing in Quebec (OR 0.49, 95% CI: 0.41, 0.59), being male (OR 0.58, 95% CI: 0.52, 0.66) and being of older age with older age being individuals greater than 30 years of age. Specifically for each incremental increase in age group, the odds of seeking support significantly decrease compared to the grouping of 18-30: ages 30-40 (OR 0.83, 95% CI: 0.71, 0.98), 40-50 (OR 0.65, 95% CI: 0.55, 0.78), 50-60 (OR 0.54, 95% CI: 0.44, 0.65), and >60 (OR 0.40, 95% CI: 0.30, 0.52). Findings are reported from the adjusted odds ratios of the model, specific to healthcare workers.

## Discussion

Many healthcare workers have reported struggling with their mental health, especially during the COVID-19 pandemic.^21^ Regardless of said struggles, there is a lack of consistency between those who experience mental health issues and those who decide to seek help. However, compared to other occupations, healthcare workers are more likely to seek mental health support. Some groups who work in healthcare, specifically people who identify as a gender other than men or women/belong to the 2SLGBTQIA+ community, and those who have physical impairments, are most likely to seek mental health support, which could be due to a multitude of factors.

Healthcare workers may be more inclined to seek mental health support than individuals belonging to other professions. This may be due to their greater knowledge and awareness of mental health issues and feelings of professional responsibility as they understand that untreated mental health issues can impact their ability to provide care.^22^ However, physicians’ mental health help-seeking can be a challenge due to fears about being labelled as unfit for the job and posing a risk to patient safety upon disclosure of mental illness. Questions may seek mental health information on physician applications for licensure and credentialing.^23,24^ When the licensing agency deems the physician as mentally unfit, they are blocked from practicing medicine. Empirical studies on Canadian physicians reveal this stigma as a contributor to mental illness among physicians and a possible barrier to reporting mental health concerns and seeking mental support through employee assistance programs.^18,23-25^ It is critical to support healthcare workers’ mental well-being by fostering safe and inclusive mental healthcare spaces.

Medical students belonging to the 2SLGBTQIA+ community reported higher levels of depression and anxiety due to the feeling of isolation and discrimination.^26^ A low sense of belonging was observed in both sexual minority and ethnic minority groups during their medical residency, which links to higher levels of burnout compared to heterosexual and White counterparts.^27^ Physicians with physical disabilities are underrepresented group which face bias and discrimination,^28^ leading to emotional exhaustion throughout their workday.^29^ As more research emerges to investigate mental health among underrepresented groups within the healthcare sector, little research is done on help-seeking behaviors and coping strategies among them.

A common barrier among healthcare professionals that may prevent them from seeking help is stigmatization, which impacts the quality of care they provide and their well-being. Studies have shown that healthcare professionals often face stigma from their peers and the broader healthcare system, which can lead to reluctance in seeking help for their mental health struggles.^30^ Systematic biases and negative attitudes towards mental health can affect healthcare workers, leading to a culture of silence and fear.^31^ Addressing this stigma is critical for creating a supporting environment that encourages healthcare providers to seek the help they need without fear of judgment or discrimination. By engaging in help-seeking behaviors, healthcare professionals may experience lower job stress and choose “thinking before acting” propensity action type. This helps to develop better strategies to deal with work stressors leading to increased job satisfaction.^32^

Older adults and men are two groups of healthcare workers identified in our study. They tend to shy away from help-seeking behaviors and prefer to handle their issues alone. Such decisions can lead to decreased mental health, occupational function, and overall poor quality of life. It is, therefore, ever so important to understand why these groups do not want to seek help as willingly as their counterparts and make recommendations as to how we believe we can aid in reducing stigma and increasing accessibility of care. The following sections will explore the context of older adults and men in an effort to explore potential reasons for the observed trends in our data.

### Older adults

Our findings highlight key factors regarding help-seeking behaviors among Canadian older adults employed in the healthcare industry. An inverse dose-response relationship was observed between age and help-seeking behavior: as age logistically increased, help-seeking for mental health decreased by approximately 10% per increase in the age category. The trend is consistent with findings from other studies on older adult’s help-seeking tendencies for mental health issues. However, the literature lacks sufficient exploration of help-seeking behaviors among older adults in the healthcare industry.

A national data from Australia reported that older doctors experienced less stigma, however, 31% of older doctors believed that mental health conditions are signs of personal weakness.^33^ This perception could explain out findings that older healthcare workers avoided asking help regarding their mental health. Among senior doctors the main barrier for seeking help was the fear of negative consequences on their career, as well as perception of mental health condition being a sign of weakness.^34^ However, Muhamad Ramzi et al.^35^report that senior doctors were more likely to ask for help rather than early-stage physicians.

The mixed results discussed across literature can be attributed to a variety of factors. Cultural and societal influences play a large role in one's beliefs towards mental health.^36^ Different cultures have varying levels of acceptance regarding mental health, which directly influences one’s likelihood to seek help. Generational differences also influence one’s beliefs.^37^ Older adults grew up in an era where mental health was less understood and stigmatized, making them less inclined to discuss their mental health experiences. Additionally, one’s geographical location impacts the accessibility and cost of healthcare. For instance, in regions where mental health services are more affordable and accessible, older adults may be more likely to seek help and vice versa. The design and methodology of studies, including sample sizes and population studies, can also contribute to differing results. Further, historical trauma or negative experiences with the healthcare system may cause distrust among certain groups, leading to lower-seeking rates compared to others.

Overall, a multitude of factors can influence one’s feelings toward help-seeking. This highlights the complexity of help-seeking behaviors and the need for practice and policy recommendations to help older adults overcome barriers to help-seeking.

### Men

Canadian healthcare workers who were men in our study were less likely to seek mental health support than Canadian healthcare workers who were women. Men are less likely to seek help for their mental health in general.^38^ For instance, men were 8% less likely to consult a general practitioner about their mental health as measured by their antidepressant use consultation compared to women.^39^ Similar quantitative studies found that men were less likely to seek help from their primary care provider than women.^38-40^ In terms of healthcare setting, female-oriented occupations are more likely to seek help rather than male-oriented occupations within healthcare industry.^41^

A variety of factors contribute to the overall lower help seeking behaviors among male healthcare workers. Male doctors are less likely to seek support directly from colleagues, and main factors were concerns to confidentiality and stigma around their distress.^42^ Female healthcare professionals reportedly had higher help-seeking behaviours.^43,44^ Among residents, men were less likely to seek professional help regarding emotional concern.^45^ Different manifestations of mental health challenges arise between men and women, particularly with differences in symptoms of mental health disorders and a lack of gender specific diagnostic criteria to effectively introduce preventative strategies for male and female healthcare professionals.^46^ The inconsistencies may be due to relatively high (20%) rate of self-prescription culture among physicians.^46^ Further, the AAMC released a review paper discussing that physicians do not treat men’s mental health effectively, and that better training on men’s symptoms and societal expectations is important.^47^ For example, women tend to experience more internal symptoms of anxiety and depression, while men experience more external symptoms such as anger towards others and substance use.^48,49^ Additionally, measurement bias (i.e. inadequate assessment of men’s experiences due to limited diagnostics and knowledge in men’s mental health) and clinicians bias may be contributing to underestimates of depression and anxiety prevalence among men.^48,49^

There are barriers to men’s help seeking behaviors for mental health conditions such as: stigma,^50^ societal inhibition of male emotional expressiveness related to masculinity^51^ lack of personal insight (i.e. knowledge) into one’s own mental health,^52^ societal expectations of men to be stoic, strong, and invulnerable impacting the downstream clinician-patient,^50,53^ gender role of being a breadwinner as a barrier in seeking mental health support (i.e. found among male nurses),^54^ and a lack of targeted mental health interventions and/or promotion of interventions created by men and for men in a gender-sensitive manner.^52^

Based on a systematic review, the following recommendations were included to improve rates of mental health help-seeking behavior among men: use of role models to convey information, educational material to improve mental health knowledge to improve identification and symptom management, motivational interviewing for behavior changes, sign posting services, and, curating and disseminating content based on positive masculine traits like responsibility and strength.^52^ Addressing stigma related to help seeking among men can be targeted by showing it as a sign of strength and bravery, a positive masculine trait.^52^ Showing cases of famous male celebrities who spoke up about their mental health struggles could also prove effective in destigmatizing help seeking.^52^ Future research should consider exploring interventions to support male healthcare workers, particularly in regard to their help-seeking behavior. However, the above recommendations can be considered and tailored to healthcare workers.^48^

### Limitations

Due to the nature of quantitative studies, some limitations must be considered. Firstly, due to the collected data being acquired through self-reporting methods, there may be a chance of response bias, recall bias, and self-selection bias, contributing to lower internal validity. To counteract this, confounding variables such as geographic region, age, and gender were weighted by MHRC authors' census data to increase internal validity. The sampling strategy was also probabilistic to ensure optimal external validity and generalizability of findings to the broader Canadian population. Furthermore, we did not conduct sample size or power calculations because we utilized an existing database rather than collecting new data. Additionally, the study did not use a standardized mental health screening tool to assess participants’ clinical levels of distress or need for mental health services. As a result, the sample may include individuals without a clinical need for mental health care, potentially influencing their health-seeking behaviors.

### Future directions

Future research should consider using longitudinal studies to examine help-seeking behaviors to allow for a more comprehensive understanding of how said behaviors evolve. Additionally, qualitative research should also be considered to explore further the reasons behind the help-seeking barriers experienced by the participants and provide deeper insights into personal and systemic factors impacting behaviors. Given our findings, identifying men and older adults as less likely to seek help, we recommend that future studies analyze these two groups exclusively. Particularly, men’s mental health research should move beyond binary comparisons between men and women to better understand the nuances of symptomatology, diagnosis and treatment application in men.^48^ Conclusively, researchers need to explore further healthcare workers’ mental health help-seeking behavior (i.e. why men and older healthcare workers are less likely to seek mental health support) as there remains a dearth in this area within the literature.^48^

## Conclusions

Our quantitative study sheds light on important factors related to mental health help-seeking behavior among healthcare workers in Canada. Among healthcare workers, factors that predict accessing mental health services include having a gender identity other than man or woman, self-identifying as 2SLGBTQIA+, and having a physical impairment. Factors that predict not accessing mental services include being Quebecois, being male, and being of older age than 30 years. Among those who did not access mental health services, barriers of the highest prevalence reported include concern about COVID-19 exposure, preference to manage issues alone, lack of perceived safety of care options available, and not knowing how or where to receive help. There is a need for the creation of targeted interventions (e.g. culturally sensitive outreach programs, gender-inclusive mental health services, peer support networks, education and training on mental health, and addressing concerns related to COVID-19 exposure) for healthcare providers that promote mental health help-seeking, particularly demographic groups identified to be less likely to seek help.
